# Comparative efficacy of intravitreal aflibercept biosimilar QL1207 versus reference aflibercept in the treatment of diabetic macular edema

**DOI:** 10.3389/fmed.2025.1662735

**Published:** 2025-10-21

**Authors:** Gaixia Zhai, Na Liu, Shaopeng Wang, Xia Zhang

**Affiliations:** Department of Ophthalmology, Zibo Central Hospital, Zibo, China

**Keywords:** aflibercept biosimilar, diabetic macular edema, foveal avascular zone, vascular density, best-corrected visual acuity

## Abstract

**Purpose:**

This study aims to comparatively evaluate the clinical efficacy of intravitreal injections of aflibercept biosimilar QL1207 and the reference aflibercept in the treatment of diabetic macular edema (DME).

**Methods:**

This retrospective study analyzed the clinical data of 80 patients (80 eyes) with DME who underwent initial treatment at our hospital’s Department of Ophthalmology between June 2023 and April 2024. Forty patients (40 eyes) received intravitreal injections of the reference aflibercept (aflibercept group). Forty patients (40 eyes) were treated with intravitreal injections of the aflibercept biosimilar QL1207 (QL1207 group). All patients received a 3 + PRN (pro re nata) treatment regimen and completed a minimum follow-up period of 12 months. Best-corrected visual acuity (BCVA), optical coherence tomography (OCT), and optical coherence tomography angiography (OCTA) were assessed before and after treatment. BCVA and central retinal thickness (CRT) were compared between the two groups at baseline and at 1-, 3-, 6-, and 12-month post-treatment. Additionally, the foveal avascular zone (FAZ) area, macular vessel density, the number of intravitreal injections required, and the incidence of adverse reactions were evaluated before and 12 months after treatment.

**Results:**

The intergroup comparison of BCVA and CRT before and after treatment showed no statistically significant differences (*p* > 0.05). After treatment, both groups showed significant improvement in BCVA and reduction in CRT compared to pretreatment values (*p* < 0.05). No statistically significant intergroup differences were observed in the FAZ area, superficial vascular density (SVD), and deep vascular density (DVD) at baseline and 12 months after treatment (*p* > 0.05 for all comparisons). Following treatment, both groups demonstrated a significant decrease in FAZ area alongside a concurrent increase in SVD and DVD compared to pretreatment values (*p* < 0.05 for all parameters). During the follow-up period, no statistically significant difference was observed in the number of intravitreal injections administered between the reference aflibercept group (3.58 ± 0.71) and the QL1207 group (3.40 ± 0.63) (*p* = 0.272). Throughout the follow-up period, no patients developed severe ocular complications, including endophthalmitis, glaucoma, cataract progression, or vitreous hemorrhage. Furthermore, no cardiovascular or cerebrovascular events were reported during the treatment period.

**Conclusion:**

Both the reference aflibercept and its biosimilar QL1207 demonstrate comparable efficacy in the treatment of DME, effectively reducing macular edema, improving BCVA, and enhancing macular perfusion status.

## 1 Introduction

Diabetic retinopathy (DR), a prevalent microvascular complication of diabetes mellitus, affects approximately 25–33% of diabetic patients. Notably, diabetic macular edema (DME) represents the primary cause of visual impairment in individuals with DR (1). DME is pathologically characterized by excessive fluid accumulation within both intracellular and extracellular retinal compartments, resulting from compromised vascular integrity and breakdown of the blood–retinal barrier (2). Recent studies have established that vascular endothelial growth factor (VEGF) plays a pivotal role in the pathogenesis of diabetic macular edema. Anti-VEGF therapeutics exert their pharmacological effects by competitively binding to VEGF, thereby reducing vascular permeability, alleviating macular edema, and consequently improving visual acuity. Currently, intravitreal anti-VEGF agents, including aflibercept, ranibizumab, faricimab, and conbercept, have become first-line treatments for DME, demonstrating significant efficacy in both visual acuity improvement and edema reduction in clinical practice (3–7). Comparative studies have demonstrated that aflibercept exhibits superior pharmacological properties relative to ranibizumab and conbercept, including a broader VEGF target spectrum (binding VEGF-A, VEGF-B, and PlGF), higher binding affinity, and extended duration of therapeutic effect. While these characteristics confer enhanced clinical efficacy in DME management (8), the significantly higher cost of aflibercept presents a notable economic barrier that may limit its widespread adoption in clinical practice, particularly in resource-constrained healthcare settings.

The expiration of patents for originator biologics, coupled with advancements in biotechnology, has facilitated the development of biosimilars, which demonstrate comparable quality, safety, and efficacy profiles to their reference products (9).

These biosimilar agents, defined as biological preparations exhibiting high structural and functional similarity to licensed biologic drugs, play a crucial role in enhancing treatment accessibility by significantly reducing medication costs while maintaining therapeutic equivalence. This development aligns with public health objectives to expand patient access to essential biologic therapies (10). Qilu Pharmaceutical Co., Ltd. has successfully developed QL1207, an aflibercept biosimilar that received approval from the National Medical Products Administration (NMPA) on 18 December 2023. This biosimilar is indicated for the treatment of neovascular age-related macular degeneration and DME, mirroring the therapeutic applications of the reference product. Clinical evaluations have demonstrated that QL1207 maintains comparable efficacy and safety profiles to the reference aflibercept while offering superior cost-effectiveness. This economic advantage significantly reduces the financial burden on patients, thereby improving treatment accessibility and adherence—a particularly crucial factor for chronic retinal conditions requiring long-term anti-VEGF therapy. To our knowledge, there is a lack of published comparative clinical trials evaluating the therapeutic equivalence of the aflibercept biosimilar QL1207 versus the reference product for DME treatment. Furthermore, the scientific community remains divided regarding the precise effects of anti-VEGF agents on macular microcirculation, with the current literature presenting conflicting evidence about their vascular impacts in the retinal microenvironment (11–13).

This study compared therapeutic outcomes between intravitreal injections of aflibercept biosimilar (QL1207) and reference aflibercept in patients with DME. Key efficacy parameters included best-corrected visual acuity (BCVA), central retinal thickness (CRT), foveal avascular zone (FAZ) area, superficial and deep vascular density (SVD, DVD) in the macular region, and complication rates. This study aims to evaluate the efficacy and safety of intravitreal biosimilar QL1207 compared to the reference aflibercept in patients with DME by assessing anatomical and functional outcomes. We hypothesize that the two agents will demonstrate therapeutic equivalence in the treatment of DME.

## 2 Methods

### 2.1 Study protocol

This study was a retrospective analysis that reviewed anonymized data collected during standard clinical care. The protocol for this analysis and the use of anonymized data were approved by the Medical Ethics Committee of Zibo Central Hospital (Approval No.: 2025 Research No. 173). Due to its retrospective nature, the committee waived the requirement for obtaining informed consent from patients.

### 2.2 Patients

This study included clinical data from 80 patients (80 eyes) with non-proliferative diabetic retinopathy (NPDR) and diabetic macular edema (DME) who were treated at the Ophthalmology Department of our institution between June 2023 and April 2024. Diagnosis was confirmed according to the 2019 American Academy of Ophthalmology (AAO) Clinical Guidelines for Diabetic Retinopathy (14). The study enrolled treatment-naïve diabetic macular edema patients aged ≥18 years with complete clinical records. Key exclusion criteria comprised (1) coexisting ocular pathologies including age-related macular degeneration, retinal vein occlusion, glaucoma, central serous chorioretinopathy, or retinal detachment; (2) history of ocular interventions such as retinal photocoagulation, intravitreal injections, photodynamic therapy, or vitrectomy; (3) presence of proliferative diabetic retinopathy; (4) significant media opacity precluding reliable optical coherence tomography (OCT) assessment; and (5) HbA1c > 7%.

### 2.3 Examination and treatment

This study is a retrospective observational study. Patient grouping was not based on randomization but was naturally formed according to the actual medication used in clinical practice. All patients were initially administered three monthly loading doses, followed by a pro re nata (PRN) treatment regimen. Intravitreal injections of either reference aflibercept (aflibercept group) or biosimilar QL1207 (QL1207 group) were administered every 4 weeks during the loading phase. Retreatment decisions were based on standardized objective criteria and were made following independent assessments by two senior retinal specialists. In cases of disagreement, a third expert was consulted for arbitration. Subsequent retreatment was determined based on predefined morphological and functional criteria: (1) OCT demonstrating a CRT exceeding 280 μm and (2) a decrease in BCVA of ≥5 ETDRS letters compared to the previous visit. Due to the retrospective nature of this study, the treating physicians were not masked to the treatment groups; however, all decisions adhered to the predefined criteria described above to minimize potential bias.

Ophthalmic examinations, including slit-lamp biomicroscopy, intraocular pressure (IOP) measurement, BCVA, fundus photography, OCT, and OCTA (Carl Zeiss Meditec AG, RTVue XR), were systematically performed before and after treatment. Retinal structural and microvascular parameters were quantitatively assessed: OCT was utilized to measure CRT, while OCTA was used to evaluate the FAZ area, SVD, and DVD. OCTA images were obtained from the 6 × 6 mm region centered on the fovea. Based on a predefined criterion for image quality, only scans with a signal strength of ≥7 or greater were retained for subsequent quantitative analysis.

To minimize selection bias inherent to this retrospective, non-randomized study, patients in the QL1207 group were matched with those in the reference aflibercept group on key baseline characteristics, including age, baseline BCVA, CRT, FAZ, and VD. After matching, the two groups demonstrated balanced baseline characteristics with no statistically significant differences.

Preoperative preparations were performed according to standard protocols for intraocular surgery. The patient and their family were thoroughly informed about the treatment process, including therapeutic objectives, surgical methodology, potential risks, and possible complications. Preoperative and post-operative care instructions were also provided.

All patients provided written informed consent for each intravitreal injection procedure. Furthermore, the use of anonymized data in this retrospective study was approved by the Ethics Committee with a waiver of study-specific informed consent.

### 2.4 Observation parameters

The BCVA (logMAR) and CRT were quantitatively assessed at baseline and at 1-, 3-, 6-, and 12-month post-injection. Glycated hemoglobin (HbA1c), FAZ area, SVD, and DVD were measured at baseline and at the 12-month follow-up time point. The number of intravitreal injections required and the incidence of adverse reactions were evaluated post-treatment.

### 2.5 Statistical analysis

All statistical analyses were performed using GraphPad Prism 9 (GraphPad Software, USA). The unit of analysis in this study was the ‘eye’. Best-corrected visual acuity (BCVA) was quantified in LogMAR units, and continuous variables were reported as mean ± standard deviation. BCVA, HbA1c, CRT, FAZ area, SVD, and DVD were assessed using a paired *t*-test or the Wilcoxon signed-rank test, depending on data distribution at baseline and post-treatment. Differences between treatment groups at each time point were evaluated using an independent sample t-test or the Mann–Whitney U-test for non-normally distributed data. Differences in the gender distribution between the two groups were assessed using Pearson’s chi-squared test.

## 3 Results

### 3.1 Baseline characteristics

In this study, the reference aflibercept group comprised 40 cases (40 eyes), including 20 male and 20 female individuals, while the biosimilar QL1207 group included 40 cases (40 eyes), with 19 male and 21 female individuals (*p* = 0.8230). The mean ages of patients in the reference aflibercept group and the QL1207 group were (48.60 ± 9.14) years and (46.70 ± 8.01) years, respectively, (*p* = 0.3277). The mean HbA1c of patients in the reference aflibercept group and the QL1207 group were (6.71 ± 0.20)% and (6.73 ± 0.17)%, respectively, (*p* = 0.5013) at baseline. The mean HbA1c of patients in the reference aflibercept group and the QL1207 group were (6.67 ± 0.16)% and (6.73 ± 0.14)%, respectively, (*p* = 0.0813) at 12 months post-treatment. Within-group comparisons showed no statistically significant changes in HbA1c levels from before to after treatment in either group.

### 3.2 Comparison of BCVA and CRT between the two groups before and after treatment

**Figure 1:**
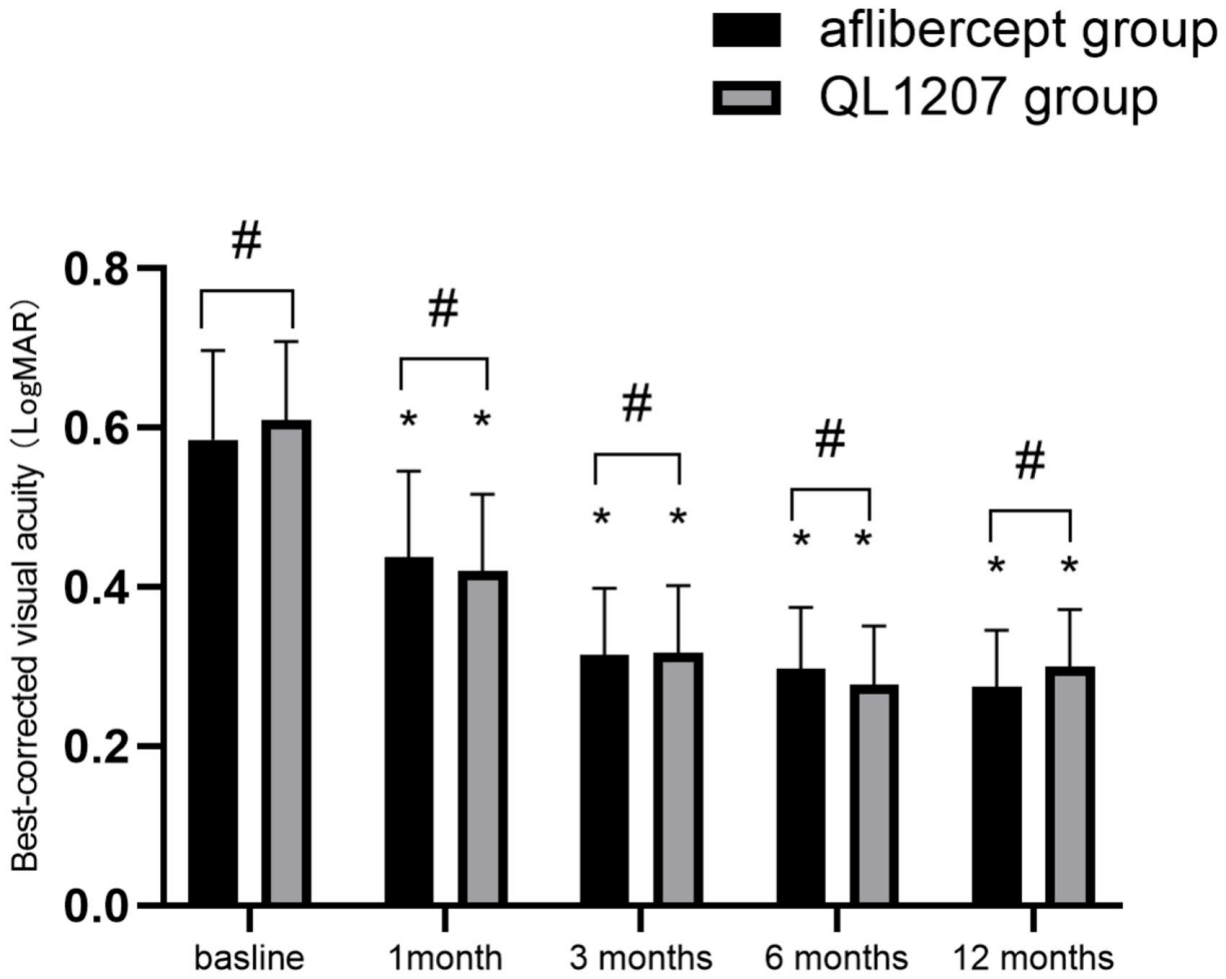
Comparison of best-corrected visual acuity between the reference aflibercept and biosimilar QL1207 groups at baseline and post-treatment (*n* = 40). * indicates a statistically significant improvement from baseline (*p* < 0.05); # (independent sample *t*-test or the Mann–Whitney U-test) denotes no significant intergroup difference between treatment groups (*p* > 0.05). Data are presented as mean ± standard deviation (SD).

**Figure 2:**
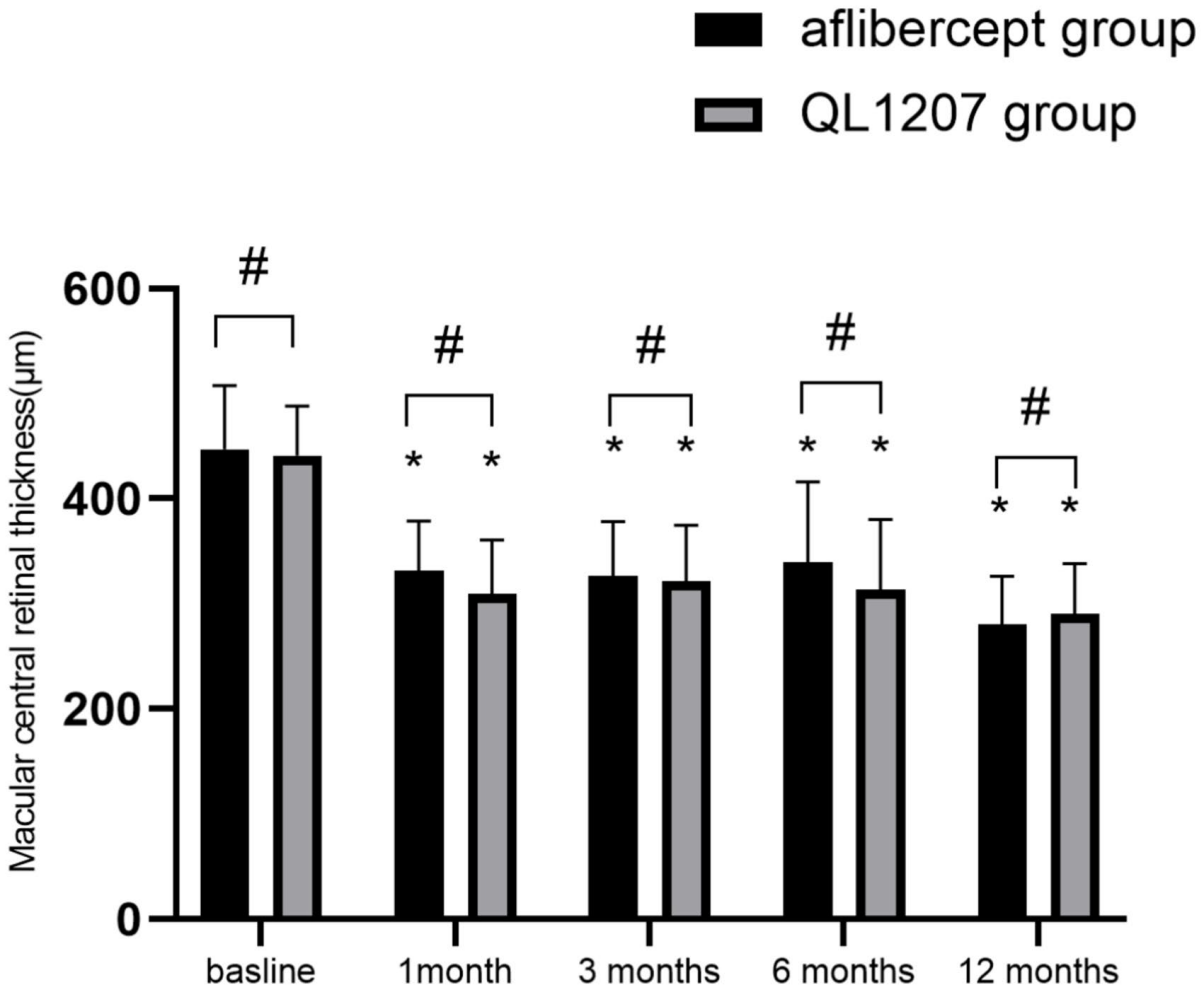
Macular central retinal thickness measurements in the reference aflibercept and biosimilar QL1207 groups at baseline and post-treatment (*n* = 40). * indicates a statistically significant reduction from baseline values (*p* < 0.05); # (independent sample *t*-test or Mann–Whitney U-test) denotes no significant intergroup difference between reference aflibercept and biosimilar QL1207 groups (*p* > 0.05). Data are presented as mean ± standard deviation (SD).

The intergroup comparison of BCVA and CRT before and after treatment showed no statistically significant differences (*p* > 0.05). After treatment, both groups showed significant improvements in BCVA and reduction in CRT compared to pre-treatment values (*p* < 0.05) (Table 1 and Figures 1, 2).

**Table 1:** Comparison of BCVA and CRT between the two groups before and after treatment.

Group	Indices (mean ± SD)	Baseline	1 month	3 months	6 months	12 months
Aflibercept (*n* = 40)	BCVA (LogMAR)	0.59 ± 0.11	0.44 ± 0.11^*^	0.32 ± 0.08^*^	0.30 ± 0.08^*^	0.28 ± 0.07^*^
	CRT/μm	447.1 ± 60.73	331.1 ± 47.58^*^	326.1 ± 52.34^*^	339.8 ± 76.66^*^	280.4 ± 45.80^*^
Biosimilar QL1207 (*n* = 40)	BCVA (LogMAR)	0.61 ± 0.10	0.42 ± 0.10^*^	0.32 ± 0.08^*^	0.28 ± 0.07^*^	0.30 ± 0.07^*^
	CRT/μm	441.0 ± 47.45	312.7 ± 49.37^*^	321.3 ± 53.43^*^	313.1 ± 67.10^*^	290.3 ± 47.87^*^

BCVA, best-corrected visual acuity; logMAR, logarithm of minimal angle of resolution; CRT, central retinal thickness; *p < 0.05 compared with baseline.

### 3.3 Comparison of FAZ area, SVD, and DVD between groups before and after treatment

**Figure 3:**
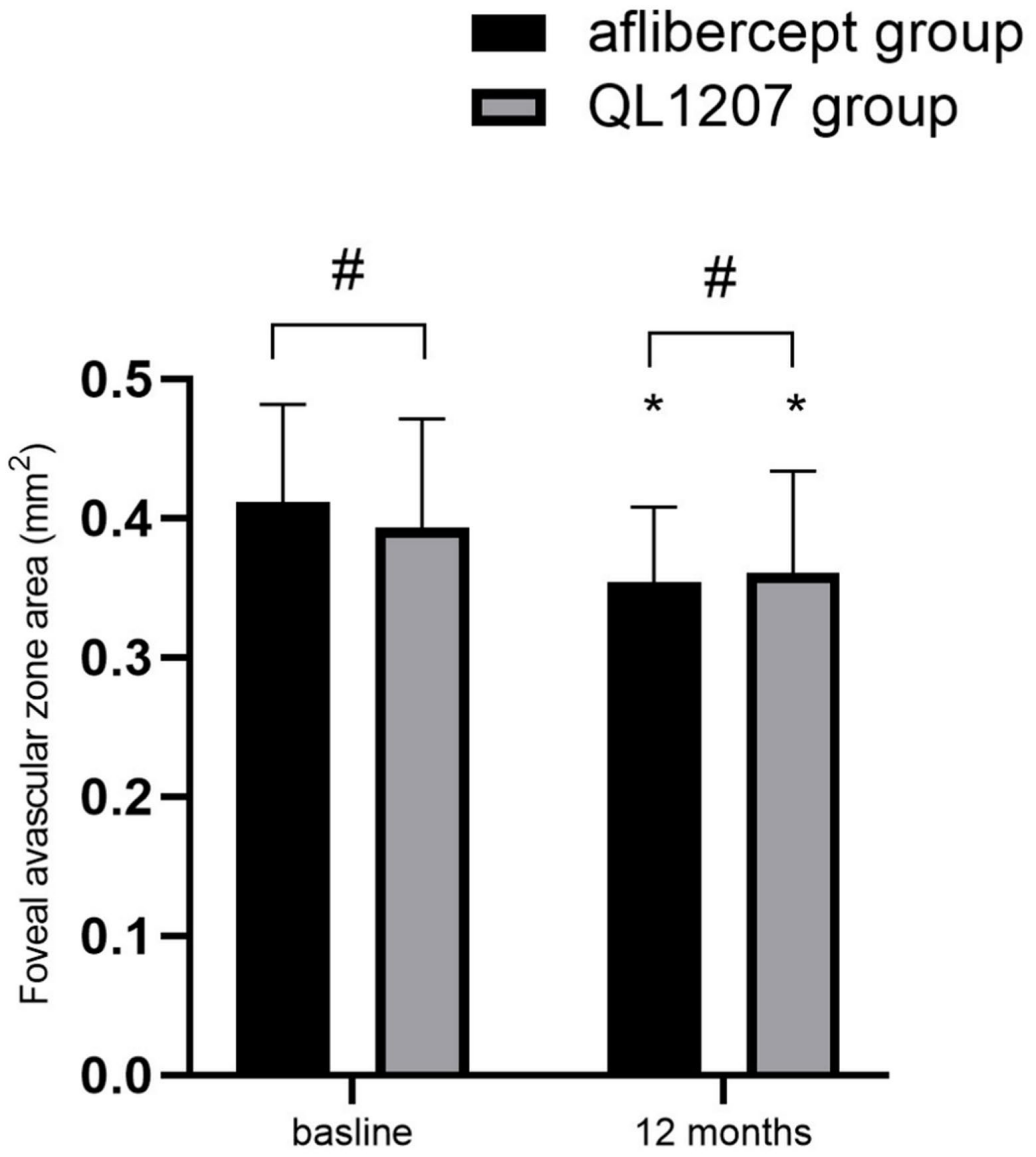
Foveal avascular zone area in the reference aflibercept and biosimilar QL1207 groups at baseline and 12 months post-treatment (*n* = 40). * indicates a statistically significant reduction from baseline (*p* < 0.05); # (independent sample *t*-test or the Mann–Whitney U-test) denotes no significant intergroup difference between reference aflibercept and biosimilar QL1207 groups (*p* > 0.05). Data are presented as mean ± standard deviation (SD).

**Figure 4:**
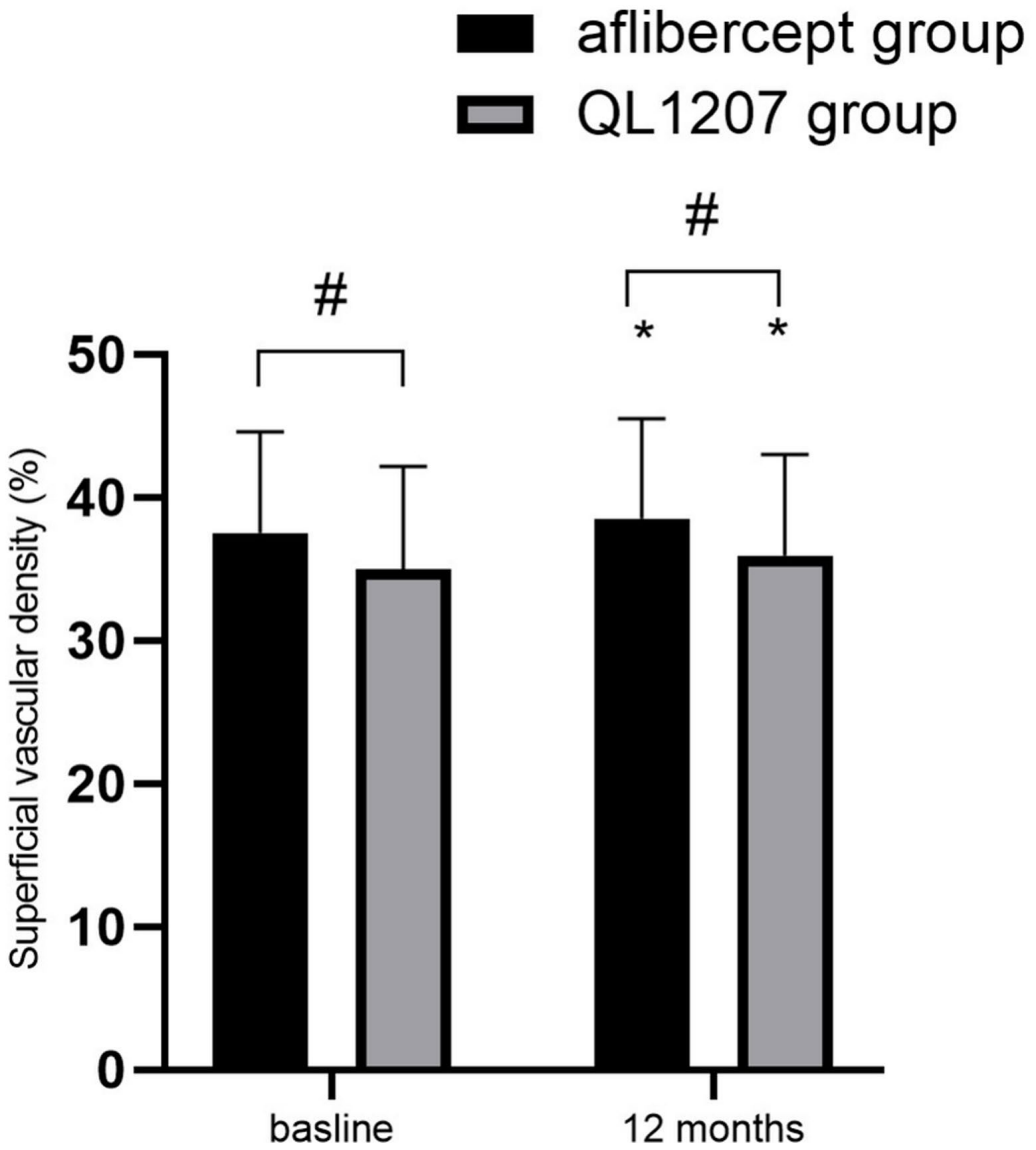
Superficial vascular density in the reference aflibercept and biosimilar QL1207 groups at baseline and 12 months post-treatment (*n* = 40). * indicates a statistically significant increase from baseline (*p* < 0.05); # (independent sample *t*-test or the Mann–Whitney U-test) denotes no significant intergroup difference between reference aflibercept and biosimilar QL1207 groups (*p* > 0.05). Data are presented as mean ± standard deviation (SD).

**Figure 5:**
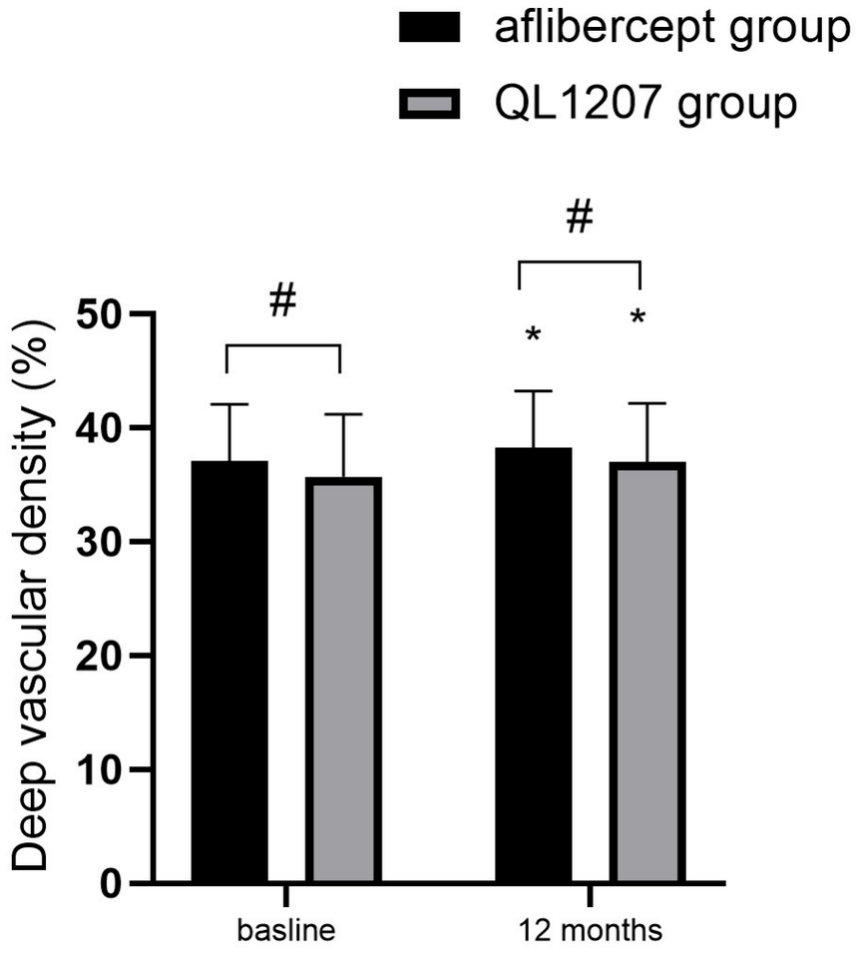
Deep vascular density in the reference aflibercept and biosimilar QL1207 groups at baseline and 12 months post-treatment (*n* = 40). * indicates a statistically significant increase from baseline (*p* < 0.05); # (independent sample *t*-test or the Mann–Whitney U-test) denotes no significant intergroup difference between reference aflibercept and biosimilar QL1207 groups (*p* > 0.05). Data are presented as mean ± standard deviation (SD).

No statistically significant intergroup differences were observed in the FAZ area, SVD, and DVD at baseline and after treatment (*p* > 0.05 for all comparisons). Following treatment, both groups demonstrated a significant decrease in FAZ area alongside a concurrent increase in SVD and DVD compared to pretreatment values (*p* < 0.05 for all parameters) (Table 2 and Figures 3–5).

**Table 2:** Comparison of FAZ area, SVD, and DVD between the reference aflibercept and biosimilar QL1207 groups before and after treatment.

Group	Indices (mean ± SD)	Baseline	12 months	*p*
Aflibercept (*n* = 40)	FAZ (mm^2^)	0.41 ± 0.07	0.35 ± 0.05	<0.0001
	SVD (%)	37.51 ± 7.10	38.51 ± 7.01	<0.0001
	DVD (%)	37.06 ± 5.01	38.27 ± 4.95	<0.0001
Biosimilar QL1207 (*n* = 40)	FAZ (mm^2^)	0.39 ± 0.08	0.36 ± 0.07	<0.0001
	SVD (%)	34.99 ± 7.18	35.94 ± 7.07	<0.0001
	DVD (%)	35.67 ± 5.50	37.02 ± 5.11	<0.0001

FAZ, foveal avascular zone; SVD, superficial vascular density; DVD, deep vascular density.

### 3.4 Comparison of the number of intravitreal injections between the two groups

During the follow-up period, no statistically significant difference was observed in the number of intravitreal injections administered between the reference aflibercept group (3.58 ± 0.71) and the QL1207 group (3.40 ± 0.63) (*p* = 0.272).

### 3.5 Complications

In the reference aflibercept group, transient intraocular pressure elevation was observed in one eye, corneal epithelial damage in two eyes, and subconjunctival hemorrhage in two eyes. In the QL1207 group, transient intraocular pressure elevation occurred in two eyes, corneal epithelial damage was observed in one eye, and subconjunctival hemorrhage in two eyes. Throughout the follow-up period, no patients developed severe ocular complications, including endophthalmitis, glaucoma, cataract progression, or vitreous hemorrhage. Furthermore, no cardiovascular or cerebrovascular events were reported during the treatment period.

## 4 Discussion

Long-term hyperglycemia in patients with diabetes mellitus has been demonstrated to impair microvascular function, inducing a cascade of oxidative stress and inflammatory responses in retinal tissues. This pathological process leads to vascular endothelial growth factor (VEGF) overexpression, which disrupts the structural and functional integrity of the blood–retinal barrier (BRB). Consequently, plasma proteins, lipids, and other intravascular components extravasate and accumulate within the retina, contributing to macular edema formation. Furthermore, macular edema exacerbates retinal ischemia and hypoxia through a negative feedback mechanism, thereby amplifying VEGF upregulation and promoting neovascularization. This vicious cycle drives disease progression, resulting in sustained pathological deterioration (15).

DME has emerged as a significant cause of vision impairment in the elderly population, garnering considerable clinical and research attention. Anti-VEGF therapy, due to its well-documented efficacy and favorable safety profile, has become the cornerstone of DME treatment. Currently, clinically available intravitreal antiangiogenic agents primarily target specific members of the VEGF family, though their therapeutic scope remains relatively restricted. For instance, ranibizumab, bevacizumab, and brolucizumab predominantly inhibit VEGF-A activity. In contrast, aflibercept exhibits a broader mechanism of action, targeting not only VEGF-A but also VEGF-B, placental growth factor-1 (PlGF-1), and placental growth factor-2 (PlGF-2) (16, 17).

Notably, aflibercept exhibits significantly higher binding affinity for VEGF165 compared to ranibizumab and bevacizumab (18). A meta-analysis of 16 randomized controlled trials (RCTs) demonstrated that intravitreal administration of aflibercept significantly improved visual acuity and effectively reduced macular edema in patients with DME (19). In 2014, the U.S. Food and Drug Administration (FDA) approved aflibercept for the treatment of DME. However, the requirement for frequent intravitreal injections of anti-VEGF agents not only imposes a substantial psychological burden on patients but also elevates their financial strain. These factors may contribute to reduced treatment adherence and an increased risk of complications (20).

In recent years, OCTA has emerged as a widely utilized, rapid, and non-invasive medical imaging modality. This technique enables three-dimensional, layer-resolved visualization of the retinal and choroidal microvasculature, facilitating the quantitative assessment of FAZ area and VD of superficial and deep retinal capillaries. Nevertheless, the impact of anti-VEGF therapy on macular perfusion and its potential role in exacerbating macular ischemia remains a subject of ongoing debate.

This study compared best-corrected visual acuity (BCVA), central retinal thickness (CRT), FAZ area, SVD, and DVD in the macular region, and complication rates in patients with diabetic macular edema (DME) before and after treatment with intravitreal injections of reference aflibercept and biosimilar QL1207 to evaluate differences in therapeutic efficacy.

The findings indicated that the intergroup comparison of BCVA and CRT before and after treatment showed no statistically significant differences (*p* > 0.05). After treatment, both groups showed significant improvement in BCVA and reduction in CRT compared to pretreatment values (*p* < 0.05). Additionally, no statistically significant intergroup differences were observed in the FAZ area, SVD, and DVD at baseline and after treatment (p > 0.05 for all comparisons). Following treatment, both groups demonstrated a significant decrease in FAZ area alongside a concurrent increase in SVD and DVD compared to pretreatment values (*p* < 0.05 for all parameters). The central retinal thickness (CRT) in the reference aflibercept group increases at the 3- and 6-month time points after initial improvement. This may be related to the treatment cycle under the PRN regimen, interindividual variability in response, or the limited sample size and suggests that future studies with longer follow-up periods are needed to validate this phenomenon.

The research results indicate that both the reference aflibercept and its biosimilar QL1207 demonstrate significant efficacy in reducing macular edema and improving BCVA in patients with DME. Additionally, both formulations exhibit comparable effects on macular perfusion status. Prior research (21, 22) demonstrated comparable therapeutic effects between the aflibercept biosimilar and the reference aflibercept for neovascular age-related macular degeneration. The findings of this study align with prior research (21, 22) and provide robust clinical evidence supporting the efficacy of QL1207 in the treatment of DME. Clinical studies have shown that the aflibercept biosimilar MYL-1701P exhibits therapeutic equivalence to reference aflibercept for diabetic macular edema management (23).

Although existing literature (24) suggests that intravitreal anti-VEGF therapy may elevate the risk of cardiovascular and cerebrovascular adverse events, no such complications were observed in our study cohort during the follow-up period. No treatment-related ocular adverse events, including endophthalmitis, vitreous hemorrhage, glaucoma, retinal detachment, or cataract progression, were observed in either treatment group during the study period.

In addition to demonstrating comparable efficacy and safety between the two treatment groups, the biosimilar QL1207 offers a considerable economic advantage, with an estimated 30% lower cost than the reference product. This price reduction has the potential to significantly reduce the financial burden on both healthcare systems and patients. Improved cost-effectiveness is a pivotal factor in promoting the integration of biosimilars into routine clinical care, as it could allow for a greater number of patients to be treated under fixed budget constraints, thereby enhancing overall access to sigh-preserving therapies.

This study has several limitations that should be acknowledged. First, its retrospective design may introduce potential biases. Second, the relatively small sample size and short follow-up duration limited the statistical power of our findings. Although statistical changes in OCTA parameters were observed, the clinical significance of their minimal magnitude remains unclear. Further research is necessary to establish a definitive relationship with visual functional outcomes. Most importantly, the long-term comparative efficacy between aflibercept biosimilar QL1207 and reference aflibercept in DME patients remains uncertain. Future research should use large-scale, prospective, long-term observational studies to address this critical question. Future prospective, randomized controlled trials with extended follow-up periods of 24 months or longer are warranted to conclusively establish the long-term therapeutic equivalence and safety profile of biosimilar aflibercept QL1207 in the management of DME.

In conclusion, both the reference aflibercept and its biosimilar QL1207 demonstrate comparable efficacy in the treatment of DME, effectively reducing macular edema, improving BCVA, and enhancing macular perfusion status. QL1207 represents a viable therapeutic option for diabetic macular edema.

## Data Availability

The datasets presented in this article are not readily available because the datasets used or analyzed during the current study are available from the corresponding author on reasonable request. Requests to access the datasets should be directed to 835076777@qq.com.
